# Role of Ferulic Acid in the Amelioration of Ionizing Radiation Induced Inflammation: A Murine Model

**DOI:** 10.1371/journal.pone.0097599

**Published:** 2014-05-22

**Authors:** Ujjal Das, Krishnendu Manna, Mahuya Sinha, Sanjukta Datta, Dipesh Kr Das, Anindita Chakraborty, Mahua Ghosh, Krishna Das Saha, Sanjit Dey

**Affiliations:** 1 Department of Physiology, Centre for Nanoscience and Nanotechnology and Centre with Potential for Excellence in Particular Area (CPEPA), University of Calcutta, Kolkata, West Bengal, India; 2 Department of Chemical Technology, University of Calcutta, Kolkata, West Bengal, India; 3 Department of Radiation Biology, UGC-DAE CSR Center Kolkata, Kolkata, West Bengal, India; 4 Cancer Biology & Inflammatory Disorder Division, IICB, Kolkata, West Bengal, India; ENEA, Italy

## Abstract

Ionizing radiation is responsible for oxidative stress by generating reactive oxygen species (ROS), which alters the cellular redox potential. This change activates several redox sensitive enzymes which are crucial in activating signaling pathways at molecular level and can lead to oxidative stress induced inflammation. Therefore, the present study was intended to assess the anti-inflammatory role of ferulic acid (FA), a plant flavonoid, against radiation-induced oxidative stress with a novel mechanistic viewpoint. FA was administered (50 mg/kg body wt) to Swiss albino mice for five consecutive days prior to exposing them to a single dose of 10 Gy ^60^Co γ-irradiation. The dose of FA was optimized from the survival experiment and 50 mg/kg body wt dose showed optimum effect. FA significantly ameliorated the radiation induced inflammatory response such as phosphorylation of IKKα/β and IκBα and consequent nuclear translocation of nuclear factor kappa B (NF-κB). FA also prevented the increase of cycloxygenase-2 (Cox-2) protein, inducible nitric oxide synthase-2 (iNOS-2) gene expression, lipid peroxidation in liver and the increase of tumor necrosis factor-alpha (TNF-α) and interleukin-6 (IL-6) in serum. It was observed that exposure to radiation results in decreased activity of superoxide dismutase (SOD), catalase (CAT) and the pool of reduced glutathione (GSH) content. However, FA treatment prior to irradiation increased the activities of the same endogenous antioxidants. Thus, pretreatment with FA offers protection against gamma radiation induced inflammation.

## Introduction

Gamma (γ) radiation is responsible for many hazardous impacts on living tissue such as DNA damage, genomic instability, apoptosis, and inflammation by the generation of reactive oxygen species (ROS). ROS primarily consisting of hydroxyl radical, superoxide anion and hydroperoxyl radical are generated by the radiolysis of water [1] and shifts the normal redox equilibrium of the cell towards oxidized state. The endogenous ROS is normally produced by lipoxygenase and NADH oxidase enzyme. It is also produced through leakage of electrons from the mitochondrial respiratory chain [2]–[11] which is scavenged by normal antioxidant defense system. γ-radiation enhances oxidative stress which is manifested by elevated ROS levels leading to a depletion of GSH pool and a reduction in SOD and catalase activity. Hence the *in vivo* antioxidant defense mechanism undergoes a threatened condition [12]. Yet another key manifestation of irradiation is oxidative stress mediated inflammation. This is primarily mediated through the activation of receptor tyrosin kinase (RTK) and redox sensitive kinases in an indirect reversible manner, which in turn phosphorylates IκBα and releases the sequestered NF-κB [13]. ROS oxidizes the essential cysteine residue with low pKa that exists as a thiolate anion at neutral pH at the active site of protein tyrosine phophatase enzyme [14]. This makes the enzyme inactive and therefore, unable to dephosphorylate the activated RTK. Activated RTK now transmits signal to the downstream pathway. Eventually, the phosphorylated NF-κB residue (p^65^) is translocated into nucleus and augments the expression of Cox-2, iNOS2, TNF-α and IL-6 inflammatory genes [15]–[18].

Several synthetic radioprotectors like lipoic acid, deoxyspergualin, cysteine, cysteamine, 2-mercaptopropionyglycine (2-MPG), amifostine [2-(3-aminopropylamino)ethylsulfanylphosphonic acid] were tested and found to be good radioprotectors [19]-[22]. However, high systemic toxicity at their optimum protective dose limits their practical application. These consequences further demand the search for less or non-toxic compounds from biological origin. These compounds include polyphenols such as hydroxybenzoic acids, hydroxycinnamic acids, anthocyanins, proanthocyanidins, flavonoids, stilbenes and lignans [23].

The FA (hydroxycinnamic acid) is commonly found in wheat, rice bran and broccoli and has a strong *in vitro* antioxidant property which includes very high DPPH radical scavenging activity, hydroxyl radical scavenging activity and nitric oxide scavenging activity. It emerges as a choice because it is cost effective, has a very promising *in vitro* antioxidant activity, higher bioavailability and less toxicity [24], [25]. Liver is known as exceedingly metabolically active organ and it reflects systemic derangement. Ionising radiation induces hepatic injury which extends as life threatening consequences [26]–[29]. Hence, liver must be protected from radiation damage. Therefore, we aimed to explore an agent that confers protection against the systemic inflammation induced by radiation. Hence we primarily attempt to examine the radioprotective role of FA against ionizing radiation mediated systemic inflammation in the Swiss albino mice. We analyzed the expression of different inflammatory markers such as Cox-2, iNOS2, TNF-α and IL-6 at different time points (6 hrs, 24 hrs, and 48 hrs post irradiation). This is the first report showing the anti-inflammatory role of FA against γ-radiation induced systemic inflammation mediated by oxidative stress.

## Materials and Methods

All Protocols including animals were approved by the Institutional Animal Ethics Committee (IAEC), Department of Physiology, University of Calcutta constituted by Committee for the Purpose of Control and Supervision of Experiments on Animals (CPCSEA), India having Permit Number: IAEC-III/proposal/SD-8/2012 dated 25.04.2012.

### Chemicals

Ferulic acid (FA), Trichloroacetic acid (TCA), Thiobarbituric acid (TBA), 5, 5′-dithio-bis (2-nitro benzoic acid) (DTNB) were purchased from Sigma (St Louis, MO, USA). Anti-Mouse monoclonal antibody against NF-κB, p-IKKα/β, Ikkα/β, p-IκBα, total-IκBα, Cox-2 were purchased from Imgenex (San Diego, CA, USA). Revert Aid M-MuLV Reverse transcriptase, oligo dT and RNase inhibitor and other chemicals required for c-DNA synthesis were purchased from Fermentas (Germany). TNF-α and IL-6 sandwich ELISA Kit were obtained from Endogen Inc. (Rockford, IL, USA). All other chemicals used were of the highest purity grade available.

### Ethics statement

This study was part of the approved proposal with certificate (IAEC-III/proposal/SD-8/2012 dated 25.04.2012.) stating that “This is to certify that the project title " Ferulic acid as a radioprotector against challenges and threats from ionising radiation -validation of molecular mechanism involving NF-kB and Nrf-2 cross talking system" under the supervision of Dr Sanjit Dey.

### Animals

Male Swiss albino mice (6–8 weeks old) were purchased from commercial source Bengal Chemical and Pharmaceuticals Ltd. (Kolkata, India) and kept under specific pathogen free conditions. Animals were maintained on a 12- hour's light-dark cycle and were fed a standard mouse diet. Mice were treated in accordance with the guidelines of IAEC and CPCSEA with appropriate temperature (23±2°C) and humidity (50±5%). The mice used had approximately equal body weight at the start of the experiments. All efforts were made to minimize suffering of the animals.

### Irradiation

Mice were irradiated using ^60^Co source in gamma chamber GC 900 (Board of Radiation and Isotope Technology, Mumbai) at Saha Institute of Nuclear Physics, Kolkata, India. Unanaesthetized mice were restrained in well-ventilated perspex boxes. They were exposed to whole body gamma radiation, at a dose-rate of 1 Gy/min and a source-to-surface distance of 77.5 cm.

### Survivability assay to determine the FA optimum dose

Mice were divided into 4 groups of 6 animals each and FA (25, 50, 75, 100 mg/kg body weight) was orally administered for 5 days. Half an hour after the last administration of FA, animals were exposed to whole body 10 Gy gamma radiation. All treated animals were observed for 30 days for any signs of radiation sickness and lethality.

### Experimental Design

Mice were selected from an inbred colony and divided into 8 groups of 8 animals each. These groups were-

**Control**: The control group received potassium phosphate buffer (0.1 M) pH 7.4 through oral gavages once in a day for five consecutive days.

**IR Group**: Mice were given potassium phosphate buffer (0.1 M) pH 7.4 for five consecutive days before exposing them to a single dose of 10 Gy ^60^Co gamma irradiation. All the irradiated animals were necropsied by cervical dislocation at 6 hours (IR6), 24 hours (IR24) and 48 hours (IR48) of post-irradiation.

**FA Group**: Mice were administered with FA (50 mg/kg body weight dissolved in 0.1 M potassium phosphate buffer) orally once a day for five consecutive days.

**FA+IR Group**: Mice were administered with FA (50 mg/kg body weight) orally for five consecutive days. Half an hour after the last dose, the animals were exposed to a single dose of 10 Gy gamma irradiation. All the animals were necropsied by cervical dislocation at 6 hours (IR6+FA), 24 hours (IR24+FA) and 48 hours (IR48+FA) of post-irradiation.

Liver was collected for the estimation of phosphorylation of IKKα/β, IκBα, nuclear translocation of NF-κB, Cox-2 protein expression, iNOS2 gene expression, lipid peroxidation (LPO), superoxide dismutase (SOD) activity, catalase (CAT) activity and reduced glutathione (GSH) level. Serum was collected for the detection of TNF-α and IL-6 by sandwich ELISA.

### Western blot assay

Western blots were performed to analyze the nuclear translocation of NF-κB, phosphorylation of IKKα/β, IκBα and Cox-2 protein expression. Liver was homogenized using a tissue homogenizer (Sono Plus, Germany) in ice-cold 0.2 mM phosphate buffer (pH 7.4) containing protease inhibitors (0.1 mM EDTA, 1.0 mM PMSF, 1 mM DTT, 0.1 mM EGTA, 0.3% NP-40 and 1 g/ml pepstatin A) to obtain a 10% tissue homogenate. 1.5 ml of the tissue homogenate was centrifuged at 12,000×*g* (30 min at 4°C) in a refrigerated centrifuge (Sorvall, USA). The supernatant was separated and re-centrifuged at 15,000×*g* (30 min at 4°C). The clear supernatant was taken for analysis of cytoplasmic fractions. The pellet obtained after the first spin (12,000×*g*) was washed thrice with PBS at 900×*g* (10 min, 4°C) and re-suspended in 0.5 ml PBS containing protease inhibitors. The suspension was centrifuged at 100,000×*g* for 1 h at 4°C using an ultracentrifuge (Hitachi, Tokyo, Japan). The supernatant was separated and used for analysis of nuclear protein. Protein concentration was determined by following the protocol of Lowry et al. [30]. Equal amounts of protein (50 µg) was loaded in each lane for 10% sodium dodecyl sulfate-polyacrylamide gel electrophoresis (10% SDS-PAGE) and transferred to a nitrocellulose membrane. The membrane was blocked overnight at 4°C with 5% bovine serum albumin (BSA) solution. Immunoblotting was done as described previously by Sinha et al. [1] using monoclonal antibody to mouse NF-κB (p^65^), phospho-IKKα/β, phospho-IBα, total IκBα, IKKα, IKKβ and Cox-2 (Imgenex, San Diego, CA, USA). β-actin and Histone 3 (H3) were used as loading control for cytosolic and nuclear extracts respectively. For each result three independent set of western blot experiments were performed using three different liver homogenate obtained from 3 different mice. Therefore, the current result is the representative of three western blot data. Immunoblots were analyzed using a model GS-700 imaging densitometer and Molecular Analyst version 1.5 software (Bio-Rad Laboratories, Hercules, CA, USA).

### Immunohistochemistry (IHC)

Immunohistochemistry was carried out on paraffin sections with anti-NF- κB (p65) antibody (Imgenex, San Diego, CA, USA). Briefly, Xylene was used to deparaffinise the sections and followed by permeabilisation by treating with 0.1% Triton X100. Then unmasking of antigens was performed by heating the sections at 90°C for 10 minutes in 10 mM citrate buffer, pH 6. After cooling at room temperature for 20 minutes each section was treated with the diluted primary antibodies overnight at 4°C. After that, the sections were then washed with PBS and incubated with appropriate dilution of secondary antibody tagged with fluorescein isothiocyanate (FITC). Nuclei were stained by using 4′, 6-diamidino-2-phenylindole (DAPI). Fluorescent signals were viewed under a microscope (Olympus IX81). To observe any nuclear translocation of NF- κB, the colour of FITC was merged with the corresponding DAPI stained nuclei. Quantification of NF- κB nuclear translocation was done by evaluating the colour intensity using ‘Image J software (1.42q version).

### Enzyme linked immunosorbant assay (ELISA)

The levels of murine serum TNF-α, IL-6 were measured using a sandwich ELISA Kit (Quantikine M; R & D systems, Minneapolis, MN55413 USA). The assay was performed as per the detailed instructions of the manufacturer.

### Polymerase chain reaction

RNA isolation was performed using TRIZOL reagent (Sigma, St. Louis, MO, USA) according to the standard protocol. 1 µg of RNA was used along with oligo (dT)_18_, 10 mM dNTP mixture, ribonuclease inhibitor, 5X reaction buffer and Revert Aid M-MuLV reverse transcriptase (Applied Biosystem, Manchester, UK) to prepare cDNA by reverse transcription. The resulting cDNA was used for Reverse Transcription-PCR (RT-PCR) using the gene specific primers for iNOS2 and 0.5 unit of Taq polymerase which yielded 496 base pair product. PCR amplification was conducted in a reaction volume of 50 µl using 2720 Thermal cycler (Applied Biosystem, UK) for 30 cycles (denaturation: at 94°C, annealing: at 58°C for 30 seconds and extension: at 72°C for 1 min and final extension at 72°C for 7 min). GAPDH expression was used as an internal control. Forward primer: 5′-GAGATTGGAGTTCGAGACTTCTGTG-3′ and Reverse primer: 5′-TGGCTAGTGCTTCAGACTTC-3′ were used. Electrophoresis was carried out using agarose gel electrophoresis and stained with ethidium bromide. The stained gels were analyzed using a model GS-700 imaging densitometer and Molecular Analyst version 1.5 Software (Bio-Rad Laboratories, Hercules, CA, USA) [31].

### Lipid peroxidation

The formation of thiobarbituric acid reactive substance (TBARS) in the homogenate was calculated using standard protocol [32]. In short the liver homogenate was mixed with TCA (15%), TBA (0.35%) and 5 N HCl followed by incubation at 95°C for 15 minutes. After cooling, all the mixtures were centrifuged and finally the absorbance of supernatant was taken at 535 nm against appropriate blank. The amount of lipid peroxidation in each sample was expressed in terms of TBARS in nanomoles/gm tissue which was estimated by using a value of ε = 1.56×10^5^ /M/cm.

### Superoxide dismutase (SOD) activity

The activity of SOD was estimated by using autooxidation of pyrogallol [33]. Auto-oxidation of pyrogallol was adjusted during calculation. In brief, 62.5 mM tris-cacodylic acid buffer was added to the liver homogenate followed by 4 mM pyrogallol. First, to monitor the auto-oxidation of pyrogallol an absorbance was taken at 420 nm. Then the absorbance of the test samples was taken at specific time intervals at same wavelength.

### Catalase activity

To study the catalase activity the absorbance of H_2_O_2_ was first taken at 240 nm then by evaluating the decrease in absorbance in the homogenate indicating the elimination of H_2_O_2_ by the action of catalase. 50 mM potassium phosphate buffer (pH 7.0), 30 mM hydrogen peroxide and 3 µl of liver homogenate were mixed in a total reaction volume of 1.0 ml. The reaction was then carried out at 20°C and only the initial linear rate of absorbance was used to estimate the catalase activity [34].

### Reduced glutathione level

Liver homogenate was treated with 0.1 ml of 25% TCA and the resulting precipitate was pelleted by centrifugation at 3900 *g* for 10 minutes. Free endogenous sulfydryl group was assayed in a total 3 ml volume by adding 2 ml of 0.5 mM DTNB prepared in 0.2 M phosphate buffer (pH 8) to 1 ml of the supernatant. The GSH reacted with DTNB and formed a yellow complex with DTNB. The absorbance was read at 412 nm [35].

### Liver function test

Serum glutamic pyruvic transaminase (SGPT), Serum glutamic oxaloacetic transaminase (SGOT), and ALP (Alkaline phosphatase) were estimated using enzymatic kit of BBI solution (73 Ty Glas Avenue Cardiff, CF14 5DX, UK,) according to the manufacturer's protocol.

### Histology

To perform the histopathological studies, small liver portions were taken and fixed in 10% formal saline solution. After appropriate fixing, the paraffin blocks of liver were prepared after dehydration followed by embedding. The 5 µm thickness paraffin sections were executed for hematoxylin and eosin stains after appropriate fixing on the slides by albumin solution. For the histopathological evaluations of liver the slides were then observed under light microscope (Olympus 207444, Tokyo, Japan) at 200× magnification. The photomicrographs were done using the Canon Power Shot S70 digital camera [36].

### Estimation of FA in plasma and liver by HPLC

Mice were administered with FA (50 mg/kg body weight) orally for five consecutive days. Animals were sacrificed after 5,15, 30 minutes and 6, 24, and 48 hours of last dose of FA administration (as in this study design, mice were exposed to radiation after 30 minutes of last dose of FA administration and sacrificed after 6, 24 and 48 hours of irradiation). Plasma and liver tissues were collected at respective time points. Liver was homogenized with 10 mM sodium phosphate buffer (pH 7.4) containing 10 mM MgCl_2_ in ice. Liver homogenate (1.5 mL) was mixed with 555 *µ*L of antioxidant solution (containing 0.2 g/mL ascorbic acid and 1 mg/mL EDTA) and 30 µL of *o*-phosphoric acid (to break the protein-phenol bond). The mixture was vortexed and centrifuged at 10,000×*g* and supernatant was used for HPLC. Solvent A consisted of 4% acetic acid in water and solvent B consisted of acetic acid/methanol/water (1∶25∶25). These solutions were eluted as follows: 0–1.5 minutes, 100% A; 1.5–10 minutes, 100% A to A: B (50∶50); 10–12 minutes, A: B (50∶50) to 100% B. The retention time for FA was about 25 minutes at 320 nm both in case of plasma and liver [1].

### Statistical analysis

The values were given as mean ± standard error of the mean (SE as error). One-way analysis of variance (ANOVA) with Tukey's post hoc test was done for statistical evaluation of the data and for the determination of level of significance in various groups of animals. In all cases, a value of p<0.05 was considered significant.

## Results

### Determination of optimum dose of FA

**Figure 1:**
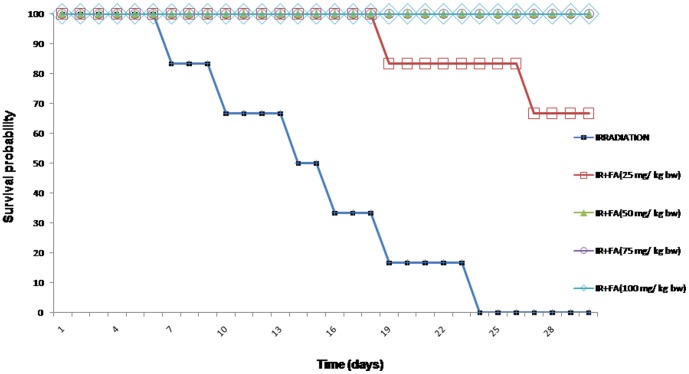
Survival probability as a function of time after exposure of Swiss albino mice to IR alone (10 Gy) or IR (10 Gy) + FA at different doses.

The irradiated animals exhibited signs of radiation sickness within 4–5 days and the first death occurred on 7^th^ day post 10Gy irradiation. All irradiated mice showed radiation sickness such as lethargy, irritability, ruffling of hair. FA control did not confer any toxic effects during the study period. FA treatment prior to irradiation caused reduced levels of radiation sickness compared to the irradiated animals. Treatment of mice with FA at a dose of 25 mg/kg body weight prior to irradiation exhibited 66.67% protection (4 out of 6 mice were alive after 30 days), whereas 50, 75 and 100 mg/kg body weight dose provided 100% protection (6 out of 6 mice were alive at the end of 30 days) in comparison to radiation control. Thus, 50 mg/kg body weight was determined as the optimum dose as it was the minimum concentration that provided 100% protection up to 30 days. On the 10^th^ day 10 Gy dose showed 33% lethality. On 15^th^ day and on the 23^rd^ day showed 66% and 100% lethality (Fig 1). No lethality was observed after FA treatment with 100 mg/kg body weight.

### Lipid peroxidation

**Figure 2:**
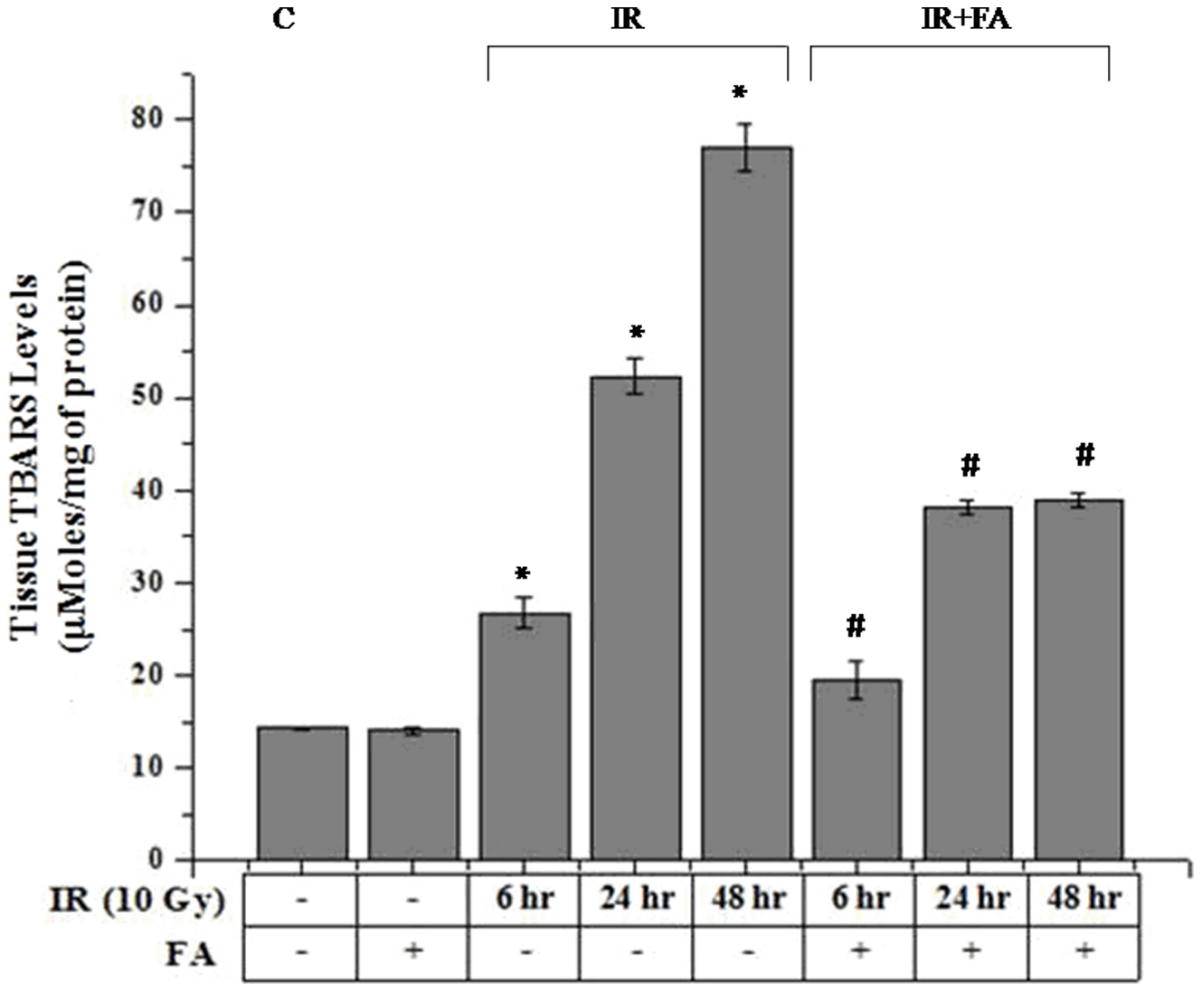
Effect of FA on radiation induced lipid peroxidation (TBARS nmol/g tissue) in mice liver. Bar 1: control group (C), Bar 2: mice treated with FA (50 mg/kg body weight) for 5 days (FA), Bar 3, 4, and 5: mice treated with 10 Gy γ-radiation and were eventually sacrificed after 6, 24 and 48 hours of radiation exposure (IR6, IR24 & IR48). Bar 6, 7 and 8: irradiated mice pretreated with FA (FA+IR6, FA+IR24 & FA+IR48). Error bars were SEM for n = 8. *p*<0.05 was considered significant. Statistical comparison: * control vs. IR, #IR vs. FA+IR.

10 Gy γ-radiation exposure to mice significantly increased the Malondialdehyde (MDA) formation which augmented higher amount of TBARS (26.78±1.59 µmoles/mg of tissue for IR6, 52.3±2.57 µmoles/mg of tissue for IR24, 76.9±1.94 µmoles/mg of tissue for IR48 groups) compared to the control group (14.3±0.23 µmoles/mg of tissue). FA pretreatment significantly ameliorated the TBARS level after radiation exposure. There was a decreased level of TBARS in treated groups (19.49±1.94 µmoles/mg for IR6+FA, 38.2±0.75 µmoles/mg for IR24+FA and 38.96±0.86 µmoles/mg for IR48+FA) obtained (Fig 2).

### GSH Level

**Figure 3:**
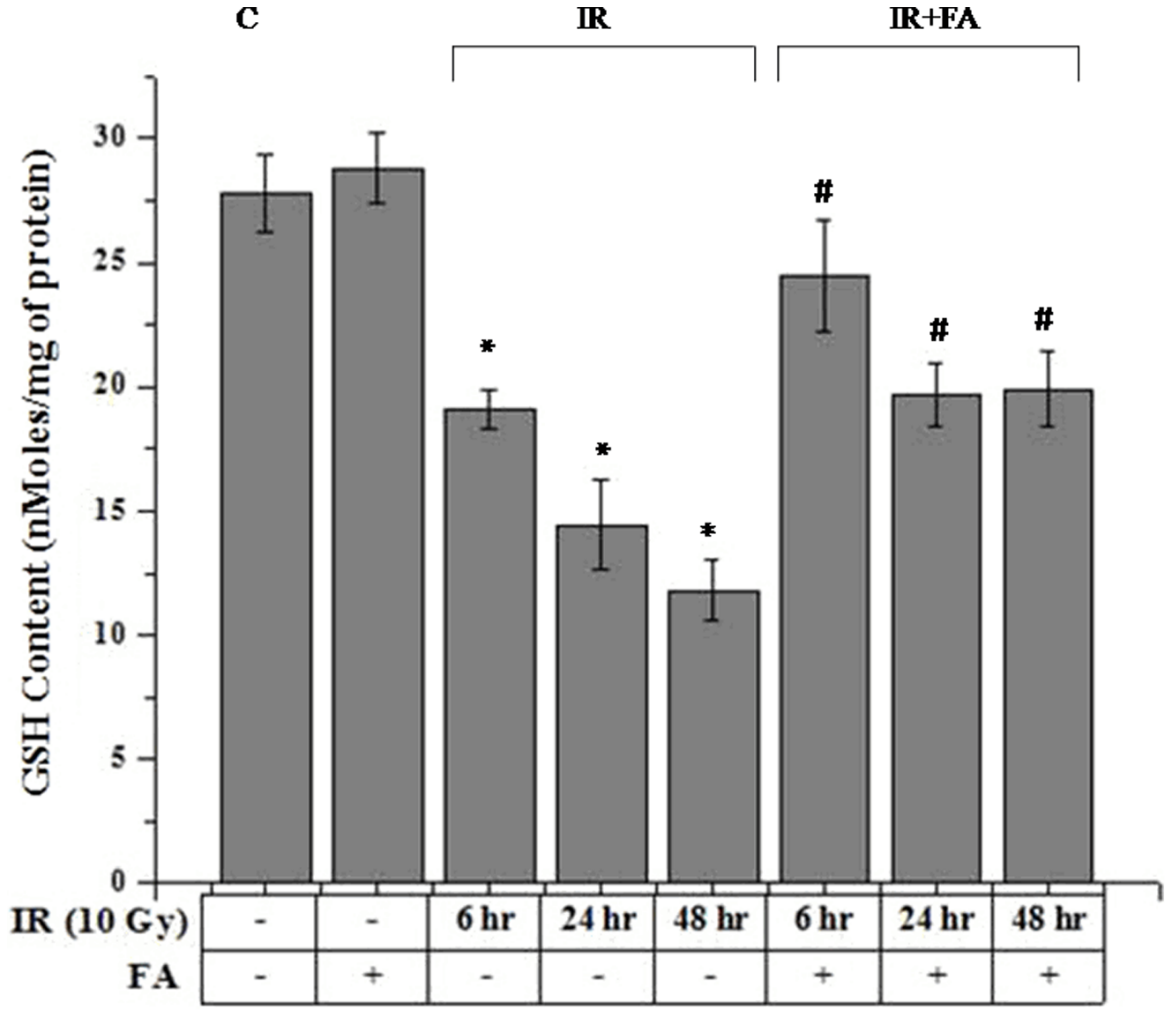
FA effect on radiation induced alteration of reduced glutathione (nmol/mg protein) level in mice. Bar 1: control group(C), Bar 2: mice treated with FA (50 mg/kg body weight) for 5 days (FA), Bar 3, 4, and 5: mice treated with 10 Gy γ-radiation and were eventually sacrificed after 6, 24 and 48 hours of radiation exposure (IR6, IR24 & IR48). Bar 6, 7 and 8: mice treated with FA plus irradiated (FA+IR6, FA+IR24 & FA+IR48). Error bars were SEM for n = 8. *p*<0.05 was considered significant. Statistical comparison: * control vs. IR, #IR vs. FA+IR.

10 Gy γ-Irradiation induced significant decrease (19.09±0.80 nmol/mg protein for IR6, 14.44±1.82 nmol/mg protein for IR24, and 11.80±1.25 nmol/mg protein for IR48) in GSH level compared to control group (27.77±1.59 nmol/mg protein). Treatment with FA resulted in insignificant increase (28.81±1.38 nmol/mg protein) in GSH level compared to the control group. Irradiated plus FA treated mice showed significant increase (24.50±2.24 nmol/mg protein for IR6+FA, 19.69±1.25 nmol/mg protein for IR6+FA, 19.91±1.51 nmol/mg protein for IR6+FA,) in GSH content compared to irradiated group (p<0.05). Therefore, hepatic glutathione content was restored after irradiation by FA pretreatment (Fig 3).

### SOD Activity

**Figure 4:**
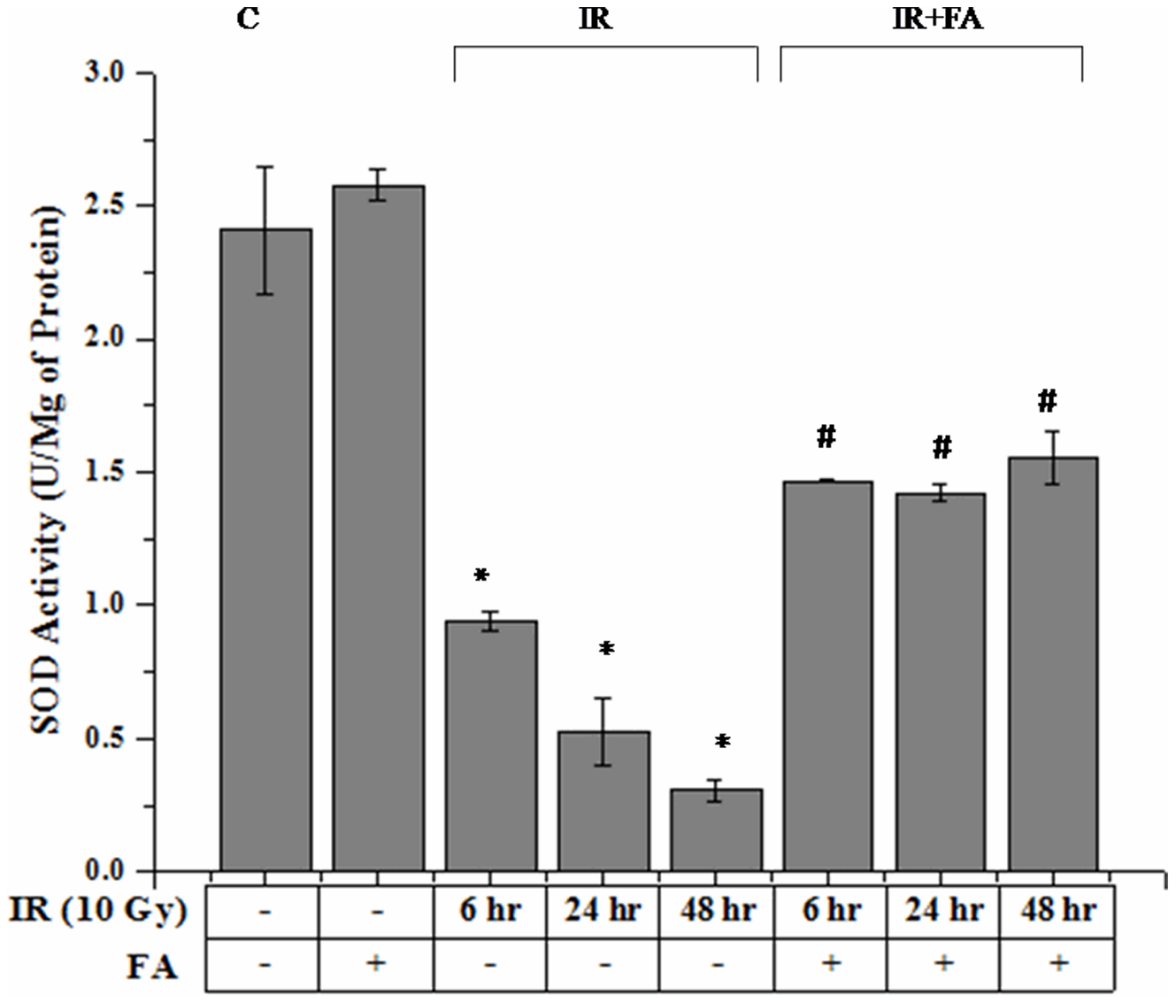
FA effect on radiation induced change in superoxide dismutase activity (U/mg protein). Bar 1: control group (C), Bar 2: mice treated with FA (50 mg/kg body weight) for 5 days (FA), Bar 3, 4, and 5: mice treated with 10 Gy γ-radiation and were eventually sacrificed after 6, 24 and 48 hours of radiation exposure (IR6, IR24 & IR48). Bar 6, 7 and 8: mice treated with FA plus irradiated (FA+IR6, FA+24 & FA+IR48). Error bars were SEM for n = 8. *p*<0.05 was considered significant. Statistical comparison: * control vs. IR, #IR vs. FA+IR.

SOD activity was significantly decreased after 10 Gy γ-radiation exposure. IR6, IR24 and IR48 groups showed an expected decrease in SOD activity (0.94±0.04 U/mg of protein for IR6 and 0.53±0.13 U/mg of protein for IR24 group, and for IR48 0.31±0.041 U/mg of protein) as compared to control group (2.41±0.24 U/mg of protein). In IR6+FA, IR24+FA, IR48+FA groups (1.46±0.003 U/mg of protein, 1.42±0.03 U/mg of protein, 1.55±0.10 U/mg of protein respectively) the activity was significantly regained by the treatment of FA before radiation exposure. FA treated group (2.57±0.06 U/mg of protein) showed SOD activity which was slightly higher than control group (Fig 4).

### Liver catalase Activity

**Figure 5:**
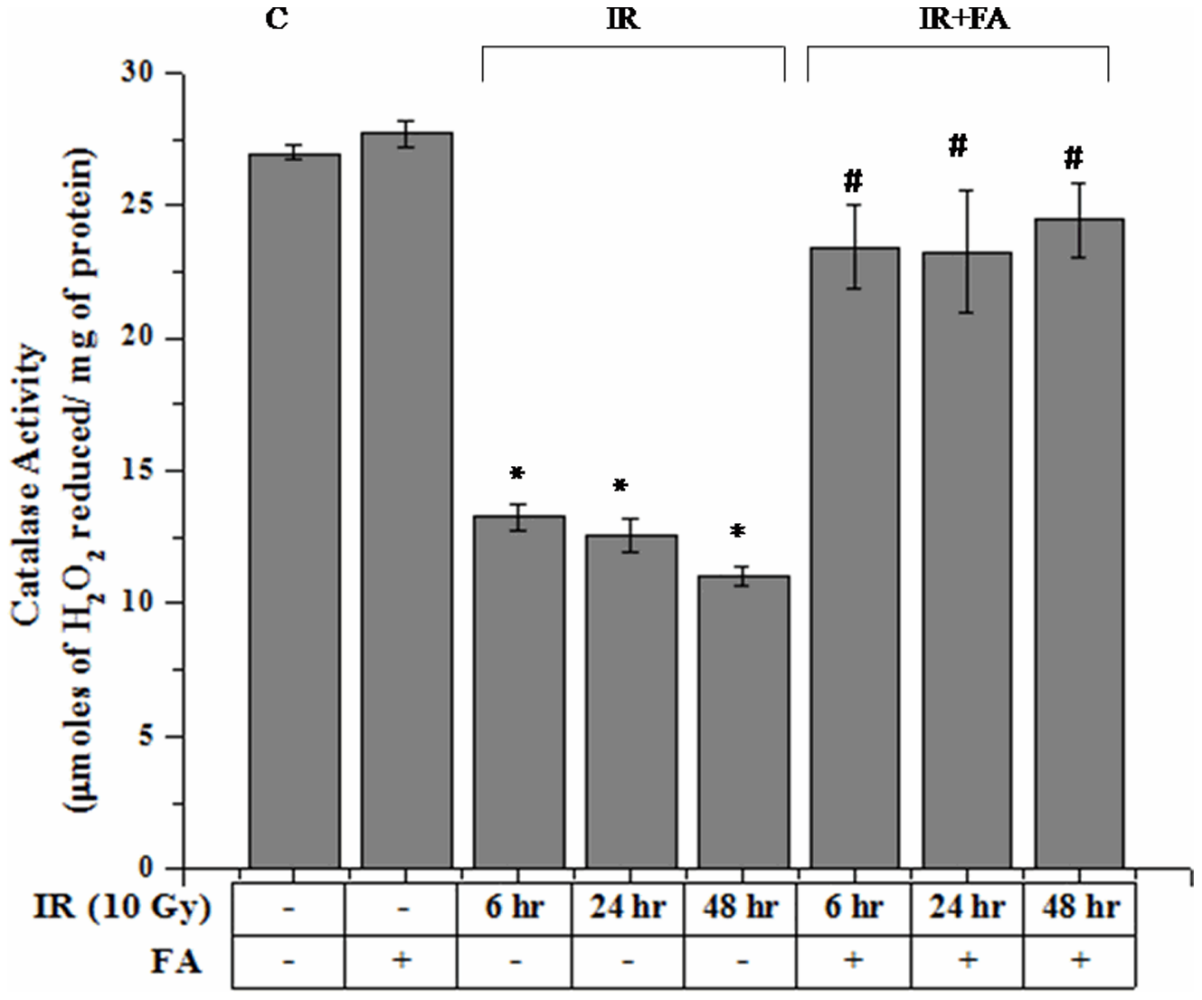
Effect of FA on radiation induced catalase activity (µmol H_2_O_2_ reduced/mg protein). Bar 1: control group, Bar 2: mice treated with FA (50 mg/kg body weight) for 5 days (FA), Bar 3, 4, and 5: mice exposed to 10 Gy γ-radiation and were eventually sacrificed after 6, 24 and 48 hours of radiation exposure (IR6, IR24 & IR48). Bar 6, 7 and 8: irradiated mice pretreated with FA (FA+IR6, FA+24 & FA+IR48). Error bars were SEM for n = 8. *p*<0.05 was considered significant. Statistical comparison: * control vs. IR, #IR vs. FA+IR.

Catalase activity was significantly decreased after 10 Gy γ-radiation exposure. IR6, IR24 and IR48 groups showed an expected decrease in catalase activity (13.29±0.49 µmol of H_2_O_2_ reduced/mg of protein for IR6 and 12.57±0.67 µmol of H_2_O_2_ reduced/mg of protein for IR24 group, and for IR48 11.05±0.32 µmol of H_2_O_2_ reduced/mg of protein) as compared to control group (26.95±0.27 µmol of H_2_O_2_ reduced/mg of protein). In IR6+FA, IR24+FA, IR48+FA groups (23.43±1.59 µmol of H_2_O_2_ reduced/mg of protein, 23.24±2.29 µmol of H_2_O_2_ reduced/mg of protein, 24.44±1.43 µmol of H_2_O_2_ reduced/mg of protein respectively) the activity was significantly regained by the treatment of FA before radiation exposure. FA treated group (27.70±0.48 µmol of H_2_O_2_ reduced/mg of protein) showed Catalase activity which was slightly higher than control group (Fig 5).

### Liver function tests (SGPT, SGOT, and ALP)

The normal functional status of liver was assessed by estimating the levels of liver enzymes ALP, SGPT, SGOT. The serum ALP (Table 1) was much elevated by gamma ray irradiation after 24 hours and 48 hours (26.22±2.94 and 35.85±4.38 respectively) compared to normal values (17.77±2.37). This elevation was reduced by treatment with FA in IR24+FA (19.99±1.72) and IR48+FA (28.38±2.34) group respectively. ALP, SGPT and SGOT levels were also increased after 6 hours of irradiation but the rise was not significant. Serum GPT levels were elevated in IR6 (4.8±0.69), IR24 (5.5±0.56) and IR48 (7.03±0.74) group due to radiation exposure and Serum GOT levels were elevated in gamma irradiated group after 24 and 48 hrs (39.5±6.26 IU/L and 48±5.27 respectively) as compared with normal levels (4.0±0.36 IU/L for GPT and 26.83±2.14 IU/L for GOT). Treatment with FA decreased this elevated levels in all FA treated groups (Table 1).

**Table 1:** Liver Function test.

	Control	FA	IR6	IR24	IR48	IR6+FA	IR24+FA	IR48+FA
ALP	17.77±2.37	10.98±1.18	19.74±1.48	26.22±2.4	35.85±4.8	17.89±1.36	19.99±1.72	28.38±2.34
SGPT (IU/L)	4.0±0.36	4.5±0.85	4.8±0.69	5.5±0.56	7.03±0.74	4.01±0.59	4.5±0.43	5.3±0.73
SGOT (IU/L)	26.83±2.41	23.66±0.76	31.62±3.68	39.5±6.26	48±5.27	27.03±3.29	27.83±3.29	42.83±1.41

Serum glutamate pyruvate transaminase (SGPT), serum glutamate oxaloacetate transaminase (SGOT) and Alkaline phosphatase (ALP) levels were determined from mice liver at different time points after radiation exposure. Radiation induced a significant increase in SGPT, SGOT and ALP level after 24 and 48 hours of irradiation. FA significantly ameliorated the radiation induced alteration of liver function.

### Radiation induced morphological/histomorphological/pathophysiological changes in liver (histology)

**Figure 6:**
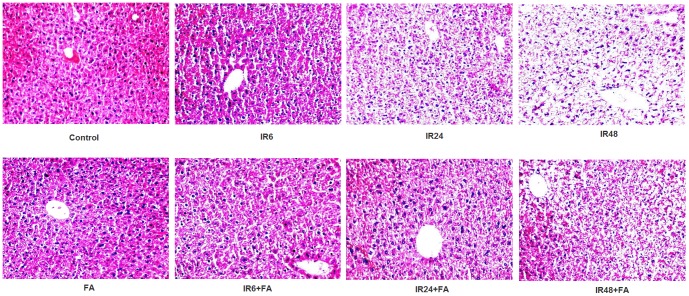
Photo micrograph from light microscope of mice liver sections. Sections were stained with haematoxylin and eosin. Magnification, X 200: Control: without any treatment, IR6, IR24 and IR48: mice irradiated with 10 Gy γ -radiation, FA: mice treated with FA (50 mg/kg bodyweight) for 5 days and IR6+FA, IR24+FA and IR48+FA: FA treated plus irradiated.

The histopathologic investigations of the liver sections showed that radiation exposure resulted in significant morphological changes compared to control; these morphological changes include oxidative stress induced damage in hepatocytes which contains pyknotic, multilobed, dense and haematoxylin rich nuclei. With the increase of time intervals hepatocytes found swelled and membranes appeared severely disrupted, sinusoidal spaces increased and disrupted nuclei of hepatocyte (IR48) which was the indication of hepatic inflammation and damage. Treatment with FA prior to irradiation prevented radiation induced inflammation and hepatic injury at respective time points (Fig 6).

### FA inhibited the radiation induced Phosphorylation of IKKα/β

**Figure 7:**
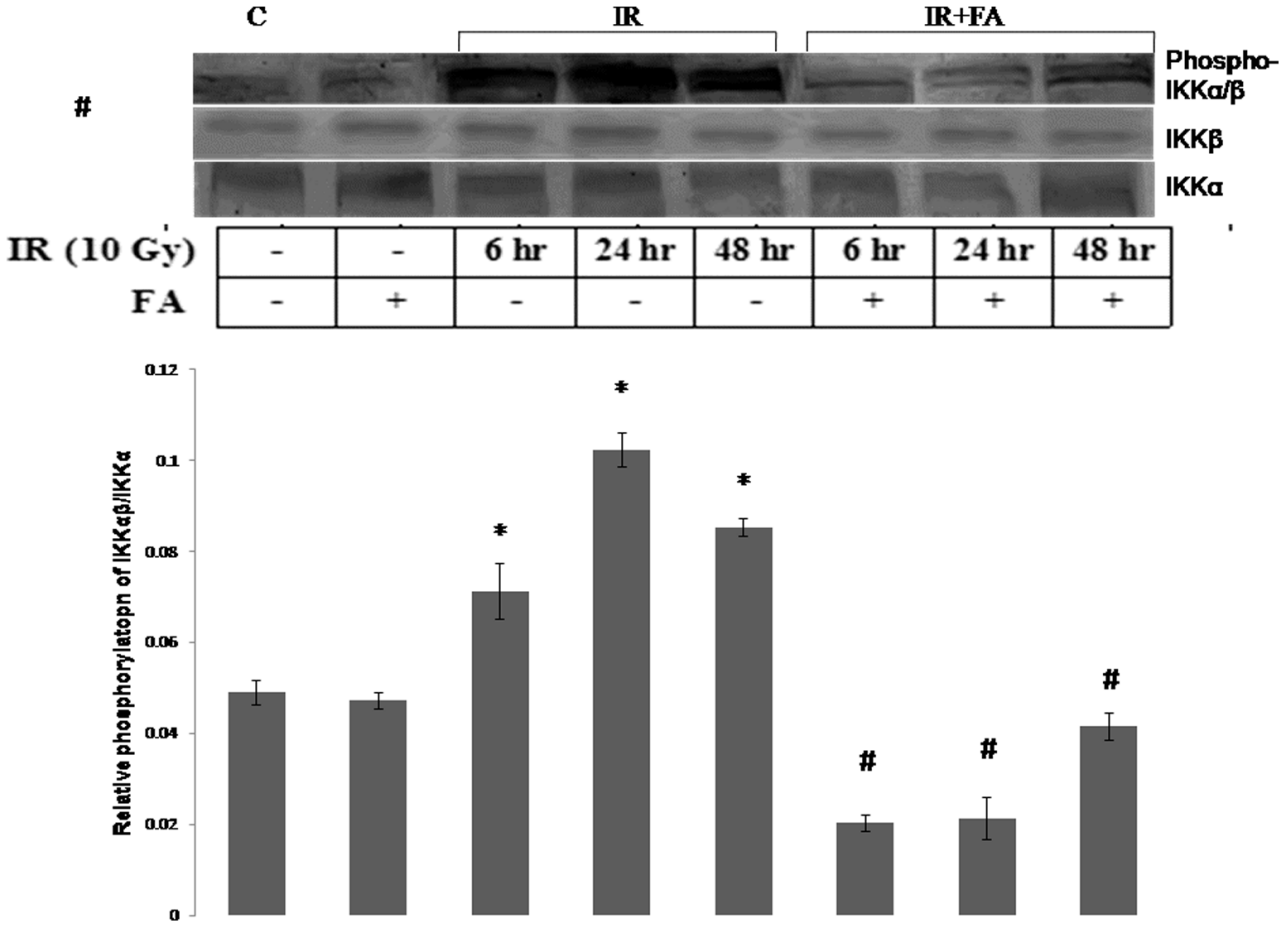
Levels of phosphorylation of IKKα/β induced by IR or IR+FA treatments. The phosphorylation of IKKα/β was determined by western blot analysis with respect to IKKα and IKKβ expression. Lane 1: control group of mice, lane 2: FA treated mice, lane 3, 4 and 5: Mice exposed to 10 Gy γ-radiation and sacrificed after 6 hours, 24 hours, and 48 hours of radiation exposure (IR6, IR24 & IR48). Lane 6, 7 and 8: mice pretreated with FA and irradiated (FA+IR6, FA+IR24 & FA+IR48). Error bars were SEM for n = 3. *p*<0.05 was considered significant. Statistical comparison: * control vs. IR, #IR vs. FA+IR.

In order to investigate the activation and phosphorylation of NF-κB pathway after irradiation and the effects of FA, we examined the phosphorylation of IKKα/β, with respect to IKKα, IKKβ level. The expression of IKKα and IKKβ remained same in all groups. FA pretreatment prevented the phosphorylation of IKKα/β in IR6+FA, IR24+FA, IR48+FA groups compared to the irradiated groups. There was no significant difference in the phosphorylation level between control group and IR+FA groups. The IR24 group showed maximum phosphorylation which was 2 fold higher than control, where as IR48 and IR6 group showed 1.2 fold, and 0.4 fold higher phosphorylation than control (Fig 7).

### Prevention of Phosphorylation of IκBα by FA

**Figure 8:**
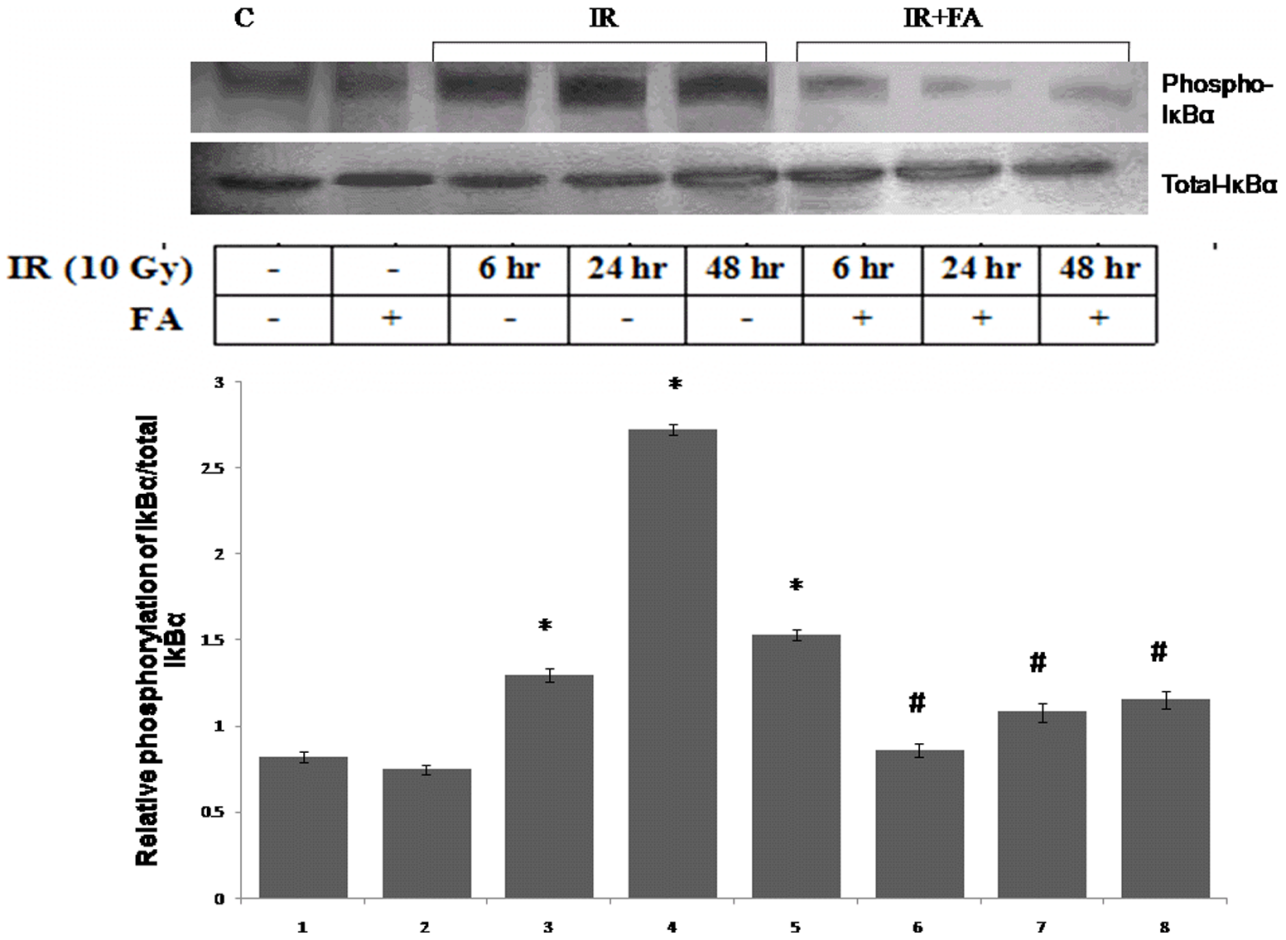
Levels of phosphorylation of IκBα induced by IR or IR+FA. The phosphorylation of IκBα was determined by western blot analysis with respect to total IκBα expression. Lane 1: control group of mice, lane 2: FA treated mice, lane 3, 4 and 5: Mice exposed to 10 Gy γ-radiation and sacrificed after 6 hours, 24 hours, and 48 hours of radiation exposure (IR6, IR24 & IR48). Lane 6, 7 and 8: irradiated mice pretreated with FA (FA+IR6, FA+IR24 & FA+IR48). Error bars were SEM for n = 3. *p*<0.05 was considered significant. Statistical comparison: * control vs. IR, #IR vs. FA+IR.

To investigate the mechanism of nuclear translocation of NF-κB, we examined the phosphorylation of IκBα in all irradiated groups as well as in treated groups of mice. There was an increase in the phosphorylation of IκBα in all irradiated groups (IR6, IR24, IR48) was observed. The IR24 group showed maximum level of IκBα phosphorylation which was 2.3 fold higher than control group. The IR48 and IR6 groups also showed higher phosphorylation with respect to control. The FA treatment prior to radiation exposure decreased phosphorylation of IκBα in IR6+FA, IR24+FA, and IR48+FA respectively as compared to irradiated groups, whereas all the groups showed similar level of total IκBα expression (Fig 8).

### Inhibition of nuclear translocation of NF-κB (p^65^)

**Figure 9:**
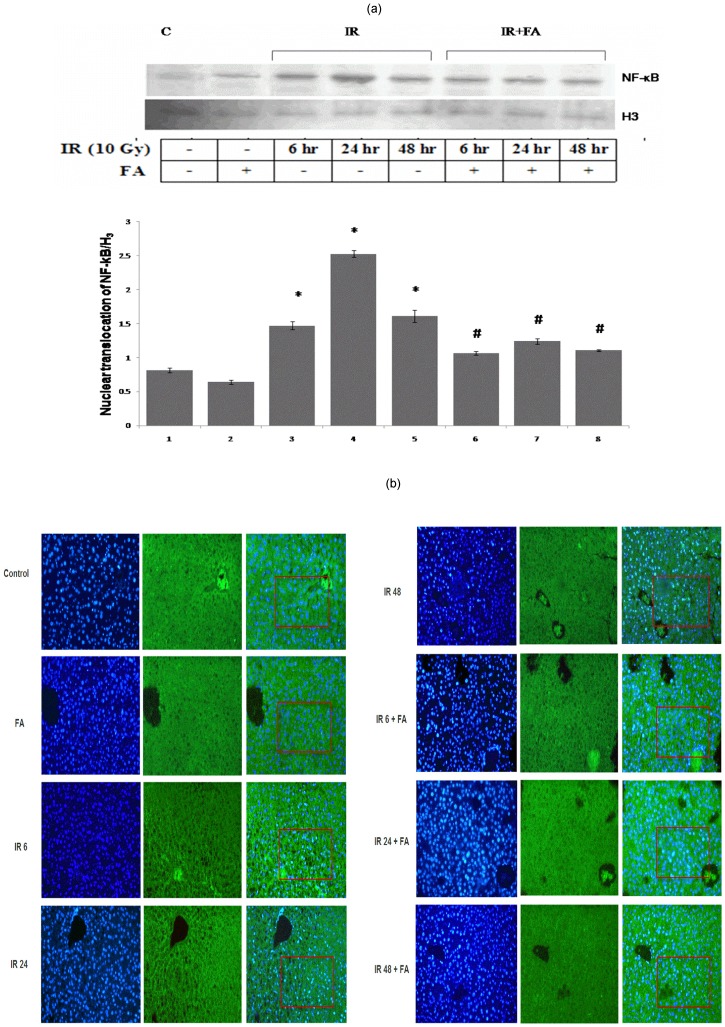
Nuclear translocation of nuclear factor kappa B (p65) after IR or IR+FA by western blot and IHC. Lane 1: control group of mice, lane 2: FA treated mice, lane 3, 4 and 5: Mice exposed to 10 Gy γ-radiation and sacrificed after 6 hours, 24 hours, and 48 hours of radiation exposure (IR6, IR24 & IR48). Lane 6, 7 and 8: mice pretreated with FA plus irradiated (FA+IR6, FA+IR24 & FA+IR48). Error bars were SEM for n = 3. *p*<0.05 was considered significant. Statistical comparison: * control vs. IR, #IR vs. FA+IR [Fig 9(a)]. NF-kB (p^65^) activation and entry into the nucleus of mice liver sections were determined by immunohistochemistry [Fig 9(b)]. Control: without any treatment, IR6, IR24 and IR48: mice irradiated with 10 Gy γ -radiation, FA: mice treated with FA (50 mg/kg bodyweight) for 5 days and IR6+FA, IR24+FA and IR48+FA: FA treated plus irradiated. NF- κB positive nuclei were estimated from the similar selected region.

The nuclear translocation of NF-κB was studied from the nuclear extract of mice liver at different time points. All irradiated groups (IR6, IR24, and IR48) showed enhanced translocation of NF-κB as compared to control which was reduced in the treated groups (IR6+FA, IR24+FA, IR48+FA). The amount of translocation of NF-κB in irradiated samples (IR6, IR24 and IR48 groups) was 0.8 fold, 2.1 fold and 0.97 fold higher than control group. This fold of rise was reduced in IR6+FA, IR24+FA, IR48+FA groups. IR24 group showed maximum level of nuclear translocation of NF-κB among all irradiated groups (Fig 9a).

### Expression of COX-2 protein

**Figure 10:**
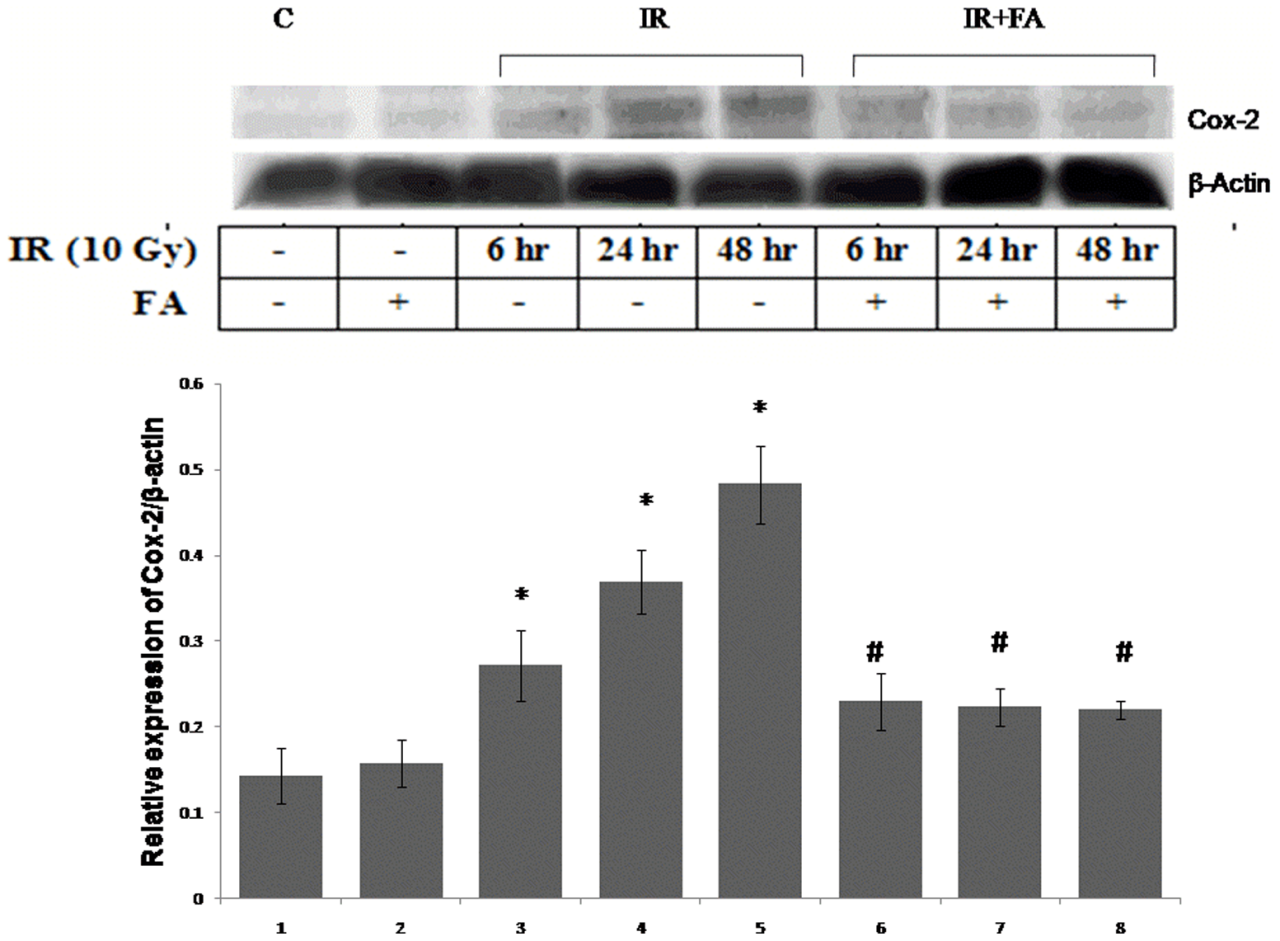
Effect of FA in the radiation induced Cox-2 protein expression. The expression of Cox-2 protein was determined by western blot analysis with respect to β-actin expression. Lane 1: control group of mice, lane 2: FA treated mice, lane 3, 4 and 5: Mice exposed to 10 Gy γ-radiation and sacrificed after 6 hours, 24 hours, and 48 hours of radiation exposure (IR6, IR24 & IR48). Lane 6, 7 and 8: mice treated with FA plus irradiated (FA+IR6, FA+IR24 & FA+IR48). Error bars were SEM for n = 3. *p*<0.05 was considered significant. Statistical comparison: * control vs. IR, #IR vs. FA+IR.

The expression of COX-2 protein was investigated from the cytosolic extract of mice liver. Mice exposed to 10 Gy gamma-radiations showed higher expression of COX-2 protein. The IR48 group showed maximum COX-2 expression with respect to control which was highest among the other irradiated groups, (Fig 10). The IR6 and IR24 group showed 0.8 fold and and 1.5 fold higher COX-2 expression than control, which was decreased in the treated groups (IR6+FA, IR24+FA, IR48+FA).

### Nuclear translocation of NF- κB (p^65^) by immunohistochemistry

To confirm the inflammatory development mediated by NF-κB, immunofluorescence method was adopted. Radiation caused nuclear translocation of NF-κB; whereas NF- κB remained localized in the cytoplasm of hepatocytes in control and FA treated group. In case of IR6 group, 12 nuclei were NF-κB positive among 79 nuclei within the selected region. Whereas, IR24 group showed maximum translocation of NF-κB (47 nuclei were positive for NF-κB among 83 nuclei) and the translocation was decreased in IR48 group (22 nuclei are NF-κB positive out of 92 nuclei in the fixed region (Figure 9b). 7 nuclei out of 101 nuclei, 18 nuclei out of 92 nuclei and 12 nuclei out of 92 nuclei were NF- κB positive in case of IR6+FA, IR24+FA AND IR48+FA group respectively. Therefore, FA pretreatment prior to irradiation decreased nuclear translocation of NF-κB. Thus, immunohistochemistry data justified the finding of immunoblot data (Fig 9b).

### FA inhibits the expression of iNOS2 gene

**Figure 11:**
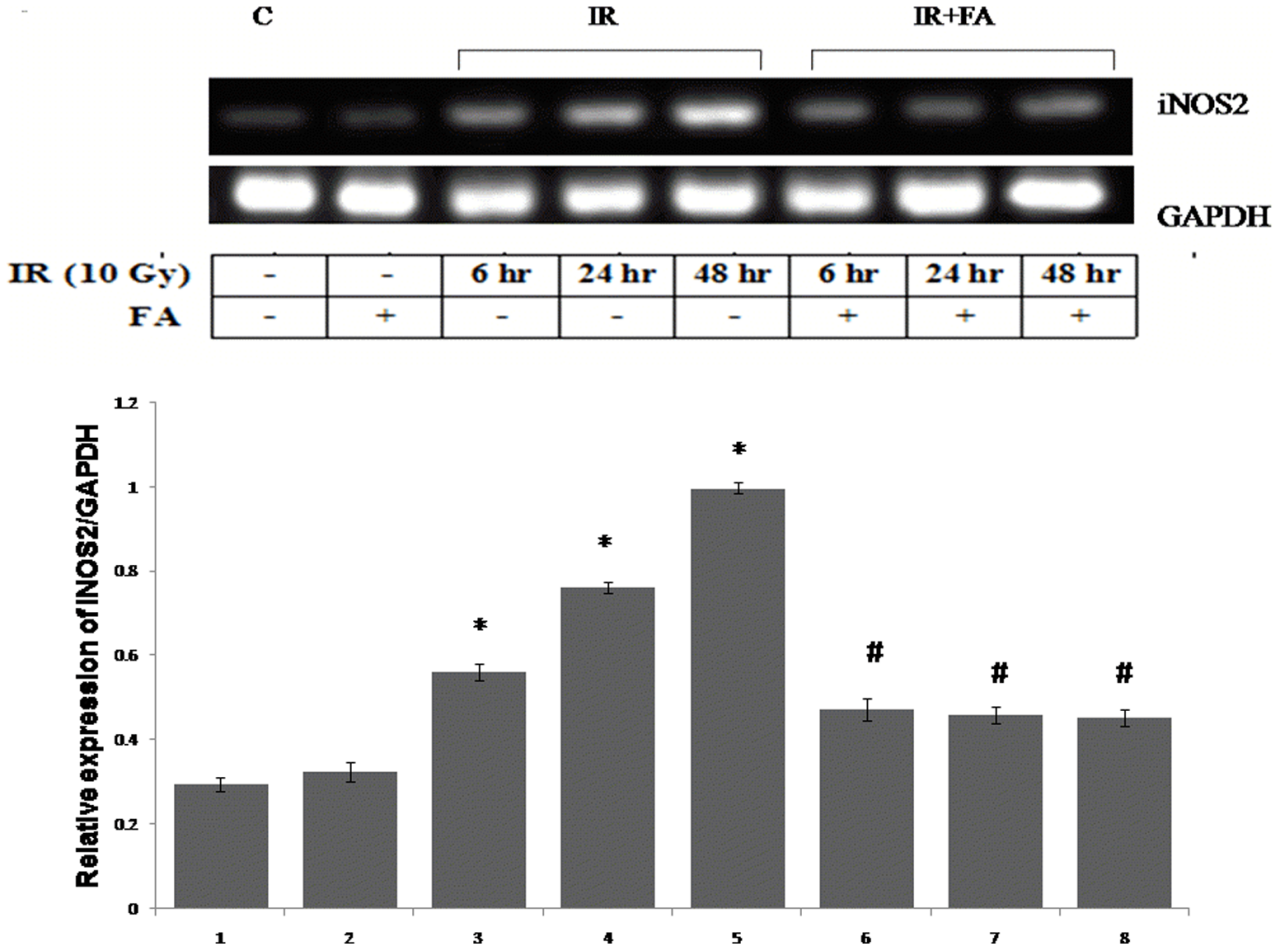
iNOS2 gene expression. iNOS2 gene expression was analysed by RT PCR. Lane 1: control group of mice, lane 2, 3 and 4: mice irradiated with 10 Gy (IR), and sacrificed after 6, 24, 48 hours of post irradiation (IR6, IR24 & IR48). Lane 5: mice treated with FA and lane 6, 7, 8: mice treated with FA plus irradiated (FA+IR6, FA+IR24 & FA+IR48). Error bars were SEM for n = 3. *p*<0.05 was considered significant. Statistical comparison: * control vs. IR #IR vs. FA+IR.

To study the radiation induced inflammatory response we investigated the iNOS gene expression, a NF-κB dependent gene product in irradiated mice at different time points. The IR48 group showed maximum gene expression of iNOS2 which was 2.3 fold higher than control, followed by IR24 and IR6 group. The FA treated group as well as all IR+FA groups showed an expected decrease in iNOS2 gene expression (Fig 11).

### FA suppresses the level of TNF-α and IL-6 in serum

**Figure 12:**
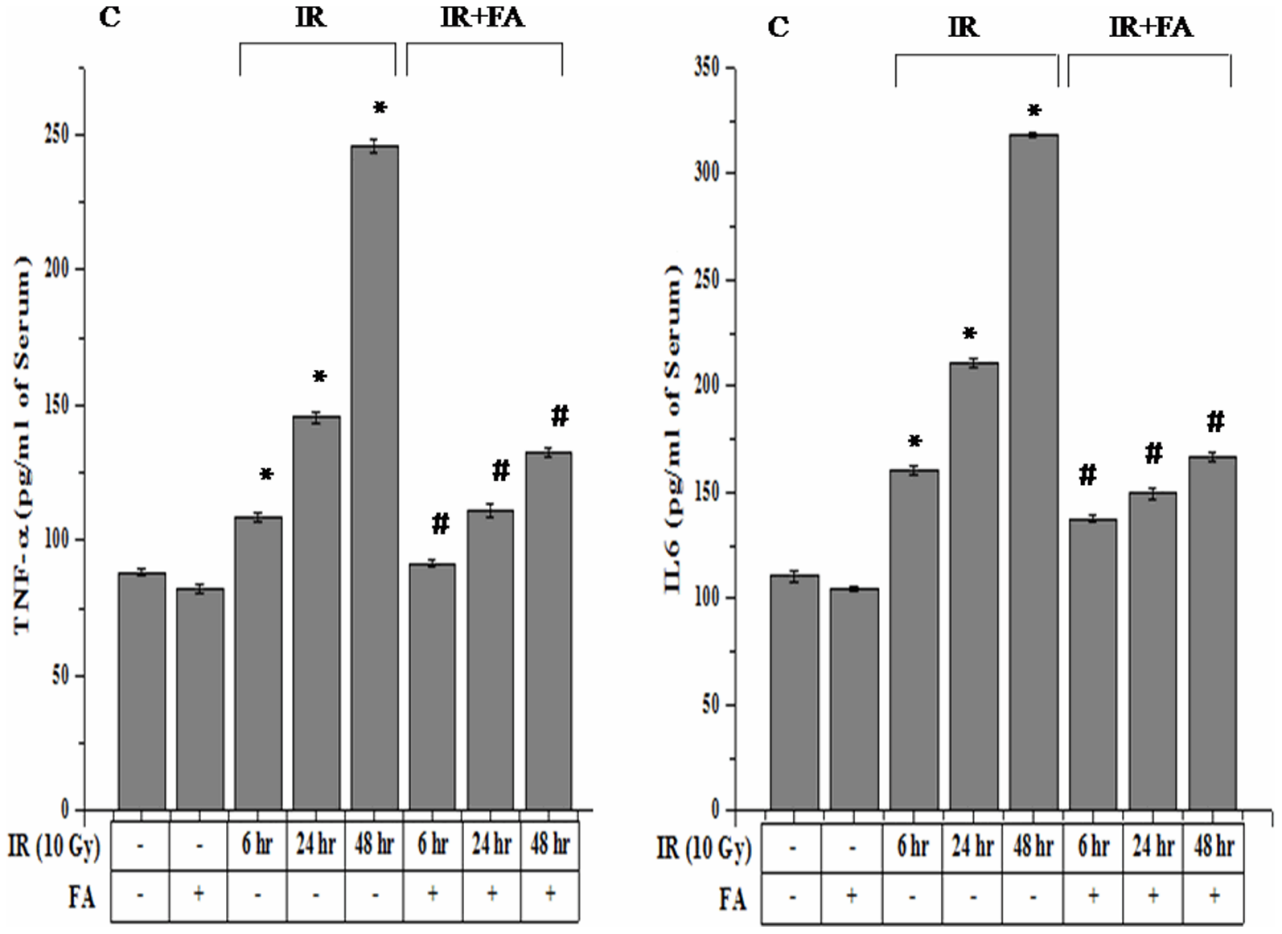
Effect of FA in the prevention of radiation induced higher level of TNF-α and IL-6 Cytokines. TNF-α and IL-6 expressions were determined by sandwich ELISA. Lane 1: control group of mice, Lane 2: mice treated with FA, Lane 3, 4, 5: mice irradiated by 10 Gy γ-radiation (IR6, IR24 & IR48). Lane 6, 7, 8: mice treated with FA plus irradiated (FA+IR6, FA+IR24 & FA+IR48). Error bars were SEM for n = 3. *p*<0.05 was considered significant. Statistical comparison: * control vs. IR #IR vs. FA+IR.

The irradiated groups showed higher expression of IL-6 and TNF-α cytokines as compared to control. However, only IR48 group showed a significant increase in IL-6 and TNF-α level (318.31±1.11 pg/ml for IL-6 and 245.85±1.29 for TNF-α) with respect to control group (110.42±2.30 pg/ml for IL-6 and 88.34±2.28 pg/ml for TNF-α). The FA treated group showed an insignificant change in IL-6 and TNF-α than control. The FA treated before radiation exposure showed a decreased level of these cytokines expression (Fig 12).

### Bioavailablity of FA in the plasma and liver

**Figure 13:**
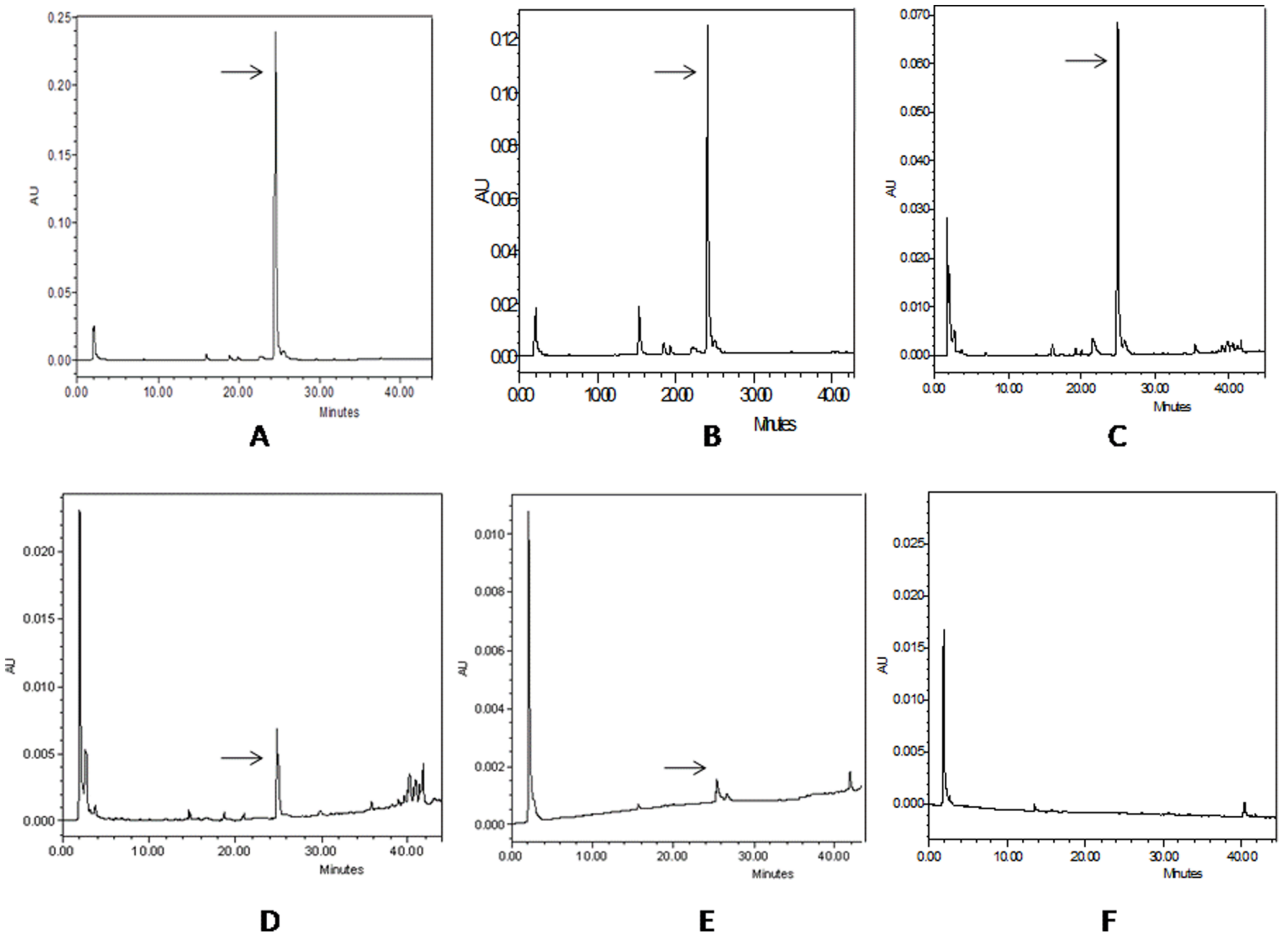
Determination of FA availability in plasma at different time points by HPLC. HPLC chromatograms of free FA in mice plasma were recorded at 320(A) after 5 minutes of FA administration; (B) after 15 minutes of FA administration; (C) after 30 minutes of FA administration; (D) after 6 hour of FA administration; (E) after 24 hours of FA administration; (F) after 48 hours of FA administration.

**Figure 14:**
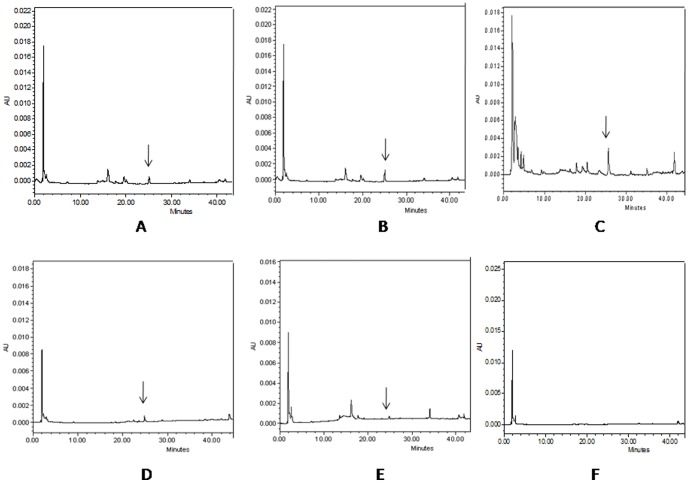
Determination of FA availability in liver at different time points by HPLC. HPLC chromatograms of free FA in mice liver were recorded at 320(A) after 5 minutes of FA administration; (B) after 15 minutes of FA administration; (C) after 30 minutes of FA administration; (D) after 6 hour of FA administration; (E) after 24 hours of FA administration; (F) after 48 hours of FA administration.

Amounts of unmetabolised form of FA in plasma and liver were calculated from the FA standard in HPLC data analysis. The concentration of FA was measured after 5, 15 and 30 minutes, and after 6, and 24 hours of FA administration. The plasma FA concentrations were 1.27 mg/ml, 0.697 mg/ml, 0.352 mg/ml, 0.028 mg/ml and 0.003 mg/ml at respective time points (Fig 13). The availability of FA in liver was also determined in liver at same time points. The concentration of FA after 5, 15 and 30 minutes and 6 and 24 hours were 11.494 µg/gm of tissue, 16.089 µg/gm of tissue, 30.486 µg/gm of tissue, 3.94 µg/gm of tissue and 0.49 µg/gm of tissue (Fig 14). After 48 hours of FA administration free FA was absent in both plasma and liver.

## Discussion

The aim of the present study was to find out the protective role of FA against gamma radiation mediated systemic inflammation. Since, liver is a major metabolic organ of the body; it encounters major challenges from the oxidative shock. Thus, the present article emphasized on the validation mechanism of the phytochemical FA which is physiologically safe but tough against the oxidative species.

In the current study, it was observed that 10 Gy gamma radiation exerted oxidative stress which was reflected by increased LPO level, decreased GSH level and decreased SOD and CAT activities. Radiation derived free radicals reacted with unsaturated lipids generating hydroperoxides. They subsequently altered lipid bilayer structure, membrane permeability, fluidity and induced lipid peroxidation [37], [38]. These cellular situations can modify low density lipoprotein (LDL) to proatherogenic and proinflammatory forms [39]. Lipid peroxidation is an indicator of the oxidative stress and inflammation. If these situations prevail, the efficacy of various defense mechanisms becomes weaker [40] and LPO products may induce mutagenesis and carcinogenesis [41]. ROS negatively regulates the intracellular concentration of GSH and the activities of enzymatic antioxidants. To combat these cellular and molecular challenges, the strategy was to strengthen the defense mechanisms by administering the exogenous substances.

The major objective of the present article was to document the inflammatory developments after radiation and its prevention by FA. There were enough proof that gamma radiation induced systemic inflammation which was reflected by (1) increased phosphorylation of IKKα/β, IκBα, (2) nuclear translocation of NF-κB by western blot and by immune fluorescence microscopic analysis, (3) increased Cox-2 protein expression, (4) induced expression of iNOS2 at mRNA level and (5) increased TNF-α and IL-6 protein expression in serum. After irradiation, increased intracellular ROS generation led to the activation of stress activated transcription factor NF-κB and AP1 (Activator Protein1). Maximum NF-κB translocation and maximum phosphorylation of IKKα/β, IkBα were observed 24 hrs after irradiation whereas IL-6, TNF-α level in serum, Cox-2 level, iNOS2 gene expression and LPO in liver was maximum after 48 hrs of irradiation. We performed the liver function tests (SGPT, SGOT and ALP) and liver histology at respective time points (6 hrs, 24 hrs and 48 hrs of post irradiation). The serum GPT, GOT and the activity of ALP were increased with the gradual increase of time after radiation exposure, indicating compromised liver function. FA pretreatment significantly ameliorated the liver function. From the liver histology data the observed explicit morphological distortions after radiation at different time points had reiterated the liver inflammation, cytokine results and liver function tests. These morphological changes include oxidative stress induced damage in hepatocytes which contains pyknotic, multilobed, dense and haematoxylin rich nuclei. With the increase of time intervals hepatocytes were found swelled and membranes appeared much disrupted, sinusoidal spaces were increased and disrupted nuclei of hepatocytes were also observed (IR48) which was the indication of hepatic inflammation and damage.

ROS, mainly H_2_O_2_ indirectly activates receptor tyrosine kinase (RTK) in a reversible manner. The cysteine residue present at the active site of the enzyme protein tyrosine phosphatase (PTP) is the target site of ROS. This enzyme is involved in the removal of phosphate group from tyrosine residue of RTK [2]. When excess amount of ROS is produced after γ-radiation exposure it eventually leads to inactivation of PTP by oxidation of specific cysteine residue at the active site of the enzyme. As a result RTK remains constitutively activated. RTK subsequently transmits signal through small GTP-binding proteins (e.g. Ras, cdc42, Rac and Rho), stimulating downstream kinases which ultimately activate MAP kinase (MAPK)-family members {ERK, JNK (also known as SAPK) and p38/RK}[42]. H_2_O_2_ increases Tyrosine phosphorylation in B cell and through the activation of PI3 kinase, which in turn augments Inositol1,4,5- tri phosphate formation leading to the opening of Ca^2+^ channel in the endoplasomic reticulum. Higher Ca^2+^ concentration in the cytoplasm initiates the proteolytic process by the activation of calmodulin and calpain [43]. Activated kinase molecules then switches on the phosphorylation cascade that ultimately phosphorylates IKKα/β, IκBα. Phosphorylated IKKα/β, IκBα then undergo proteosomal degradation, generating free NF-κB (p^65^) which translocates into the nucleus.

NF-κB binds to specific sequences in the promoter region of target genes which in turn enhance a variety of proinflammatory molecules expression such as tumour necrosis factor α (TNF-α), interleukin 1b (IL-1b), IL-6, IL-8, Cox-2 [15]-[18] and intercellular adhesion molecule 1 (ICAM-1). Radiation induced increase of ICAM-1, VCAM, junctional adhesion molecule (JM-1) genes expression were also reported in rat liver but PECAM-1 gene expression remained unchanged. This indicates that gene expression of adhesion molecules was controlled by the expression of acute phase cytokines, secreted after whole body irradiation [44].

**Figure 15:**
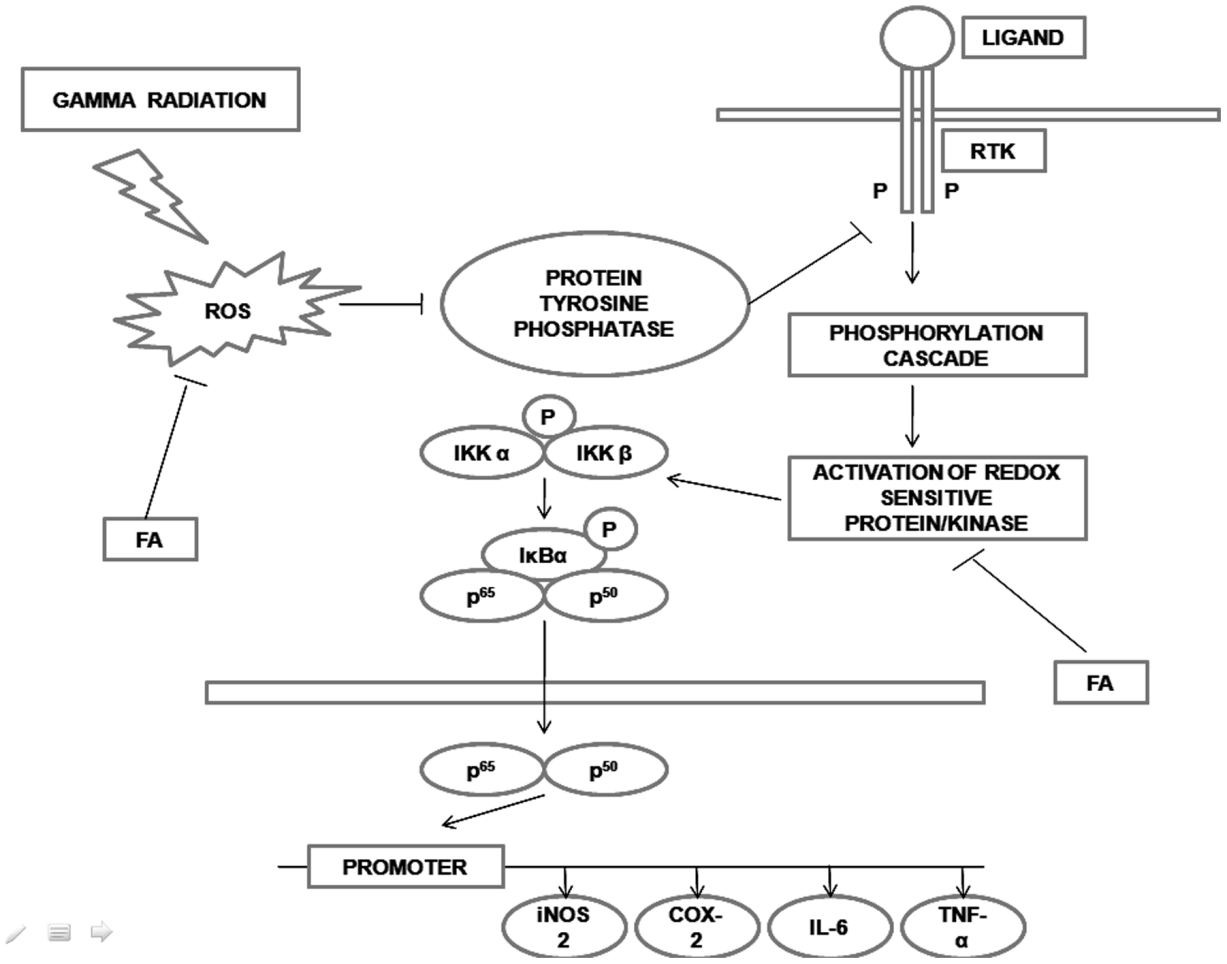
Schematic diagram of proposed mechanism. ROS produced by gamma radiation deactivated the protein tyrosine phosphatase enzyme thereby activating RTK in an indirect, reversible manner. ROS also changed the intracellular redox potential and activated redox sensitive protein/kinase. At the end of this signaling pathway the redox sensitive transcription factor NF-κB translocated to nucleus and activated its dependent inflammatory genes. FA scavenged the ROS, thereby inhibiting the activation of RTK and translocation of NF-κB to nucleus and subsequent inflammatory gene expression.

FA is a potent antioxidant which is known to protect against cancer, cold, flu, influenza, skin aging, and muscle wasting. FA can accept an extra electron from superoxide radicals therefore, preventing the free radical chain reaction and ROS generation [45]. In our study, FA treatment prior to irradiation significantly decreased the lipid peroxidation and increased the activity of SOD, Catalase and GSH level at different time points (6 hrs, 24 hrs and 48 hrs). As the FA scavenged most of the ROS, less ROS can interfere with membrane lipid. Thus less LPO was produced. As FA could restore the intracellular redox balance therefore, redox sensitive kinase activation was less. Thus, the phosphorylation of IKKα/β, IκBα decreased and less translocation of NF-κB into the nucleus was observed at different time points (Fig 9a and 9b) by immuno blot and immunohistochemistry. The level of proinflammatory cytokines such as IL-6 and TNF-α in serum, expression of iNOS2 gene at mRNA level and Cox-2 protein were also decreased in FA pretreated group and in all IR+FA groups. NF-κB, a redox sensitive transcription factor is mainly responsible for the expression of such proinflammatory genes. Since the amount of NF-κB translocation in presence of FA was diminished into the nucleus, subsequently less expression of above mentioned genes were observed after 6, 24 and 48 hrs of irradiation. The proposed mechanism is been shown in Fig 15. To investigate the presence of FA after 6, 24 and 48 hrs of last dose administration, we studied bioavailability of free FA by HPLC. We found FA in plasma up to 24 hrs in its free form, as also in liver after last dose. This physiological level of FA in free form explains that it continuously can confer protection against physicochemical factors generated by γ- irradiation. After 48 hrs of last dose there was no free FA was present either in plasma or in liver. But IR48+FA group still showed protection against γ-radiation. This happened because FA that was initially present in the system to scavenge the ROS, which decreased the oxidative stress and subsequent NF-κB translocation, inflammatory gene expression. Moreover, FA was metabolized within the biological system into different form. Thus, the current study is a comprehensive documentation in favour of cellular physiological mechanism with a cascade of experiments, validating the cellular stress ameliorating role of FA in after radiation exposure. Thus, FA, a major bioactive phytochemical, provides prevention against gamma radiation mediated systemic inflammation and thus qualify as a potent radioprotector.
